# Draft Genome Sequence of *Campylobacter jejuni* Bf, an Atypical Strain Able To Grow under Aerobiosis

**DOI:** 10.1128/genomeA.00120-16

**Published:** 2016-04-07

**Authors:** Vicky Bronnec, Nabila Haddad, Stéphane Cruveiller, Mathieu Hernould, Odile Tresse, Monique Zagorec

**Affiliations:** a Institut National de Recherche Agronomique, UMR1014, Secalim, Nantes, France; b LUNAM Université, Oniris, Nantes, France; c CNRS-UMR 8030 and Commissariat à l’Énergie Atomique CEA/DSV/IG/Genoscope LABGeM, Évry, France

## Abstract

In this study, we describe the draft genome sequence of a *Campylobacter jejuni* clinical isolate issued from a French patient suffering from severe campylobacteriosis. This atypical strain is characterized by an unusual resistance to oxygen and the ability to grow under an aerobic atmosphere, a characteristic as-of-yet unique to this species.

## GENOME ANNOUNCEMENT

*Campylobacter jejuni* is among the leading causes of foodborne bacterial enteritis in the world and the most frequently reported in foodborne illness in the European Union since 2005, with 214,779 confirmed cases of human campylobacteriosis in 2013 (1). This zoonosis, which is steadily increasing, is transmitted to humans mainly through the consumption of contaminated food, particularly poultry meat (2). Although *C. jejuni* behavior and virulence have largely been studied, this pathogen remains uncontrolled in the food chain. *C. jejuni* belongs to the *Epsilonproteobacteria* and is microaerophilic and capnophilic. It can multiply in a variety of ecological niches, and it survives under harsh environmental conditions (3, 4). The *C. jejuni* Bf clinical isolate presents an unusual resistance to oxygen and can grow under an aerobic atmosphere, characteristics that were not previously reported in the *C. jejuni* species (5).

The genomic DNA was sequenced using the Illumina HiSeq 2000 sequencing system, generating ~25,000,000 reads that were assembled *de novo* with the Velvet software (6). Contig sequences were mapped on the reference genome sequence of *C. jejuni* NCTC 11168 (accession no. NC_002163.1) (7, 8). Misassembled regions were checked manually, and PCR amplification products were sequenced for gap filling.

The genome of *C. jejuni* Bf consists of a 1,506,810-bp circular chromosome, with an average G+C content of 30.44%. Annotation performed on the MicroScope platform (MaGe) (9) detected 1,635 coding sequences (CDSs) and 22 tRNAs. The genome contains three rRNA operons. No plasmid-related sequence was noticed.

The comparison of the *C. jejuni* Bf draft sequence with 32 complete and 19 draft *C. jejuni* genome sequences did not reveal any gene unique to *C. jejuni* Bf. However, some CDSs presented putative point mutations or deletions susceptible to affect several functions. Among those, CJBOF_v2_160020 and CJBOF_v2_160021 may result from a cleavage of the *cj0309c* gene from the reference strain *C. jejuni* NCTC 11168, encoding a multidrug resistance transporter. Interestingly, the *C. jejuni* Bf *oorD* gene encoding one of the 2-oxoglutarate oxidoreductase (OOR) subunits was mutated at position 187, resulting in an Ile63Thr mutation located within the second (4Fe-4S) cluster. This may affect the structure and therefore the activity of OOR. In *Helicobacter pylori*, OOR catalyzes the formation of succinyl-coenzyme A (CoA), an intermediate of the tricarboxylic acid (TCA) cycle (10, 11), and it seems to provide NADPH with a respiratory donor electron (11). In *H. pylori*, the oxygen-labile OOR enzyme contributes to the microaerophilic phenotype (11). *C. jejuni* has a TCA cycle similar to that of *H. pylori*, and the same *oorDABC* operon is observed. Therefore, a mutation in the *C. jejuni* Bf *oorD* gene may result in an altered phenotype regarding the oxygen metabolism in this strain, although an increased sensitivity to oxygen would be expected. To conclude, the aerotolerance of *C. jejuni* Bf could not clearly be attributed to gene acquisition or mutation accumulation. Modifications at the transcriptional, posttranscriptional, translational, or posttranslational level might therefore be hypothesized to explain the atypical phenotype of this strain.

### Nucleotide sequence accession numbers.

This whole-genome shotgun project has been deposited in ENA under the accession numbers FCEZ01000001 to FCEZ01000095. The versions described in this paper are the first versions.
